# Serological surveillance on potential *Plasmodium vivax* exposure risk in a post-elimination setting

**DOI:** 10.3389/fcimb.2023.1132917

**Published:** 2023-03-09

**Authors:** Fang Huang, Yanwen Cui, Zhuoying Huang, Siqi Wang, Shigang Li, Xiangrui Guo, Xiang Guo, Zhi-Gui Xia

**Affiliations:** ^1^ Institute of Immunization, Shanghai Municipal Center for Disease Control and Prevention, Shanghai, China; ^2^ Division of Tuberculosis Control and Prevention, Shanghai Pudong Center for Disease Control and Prevention, Shanghai, China; ^3^ National Institute of Parasitic Diseases, Chinese Center for Disease Control and Prevention, Shanghai, China; ^4^ Department of Malaria, Chinese Center for Tropical Diseases Research, Shanghai, China; ^5^ NHC Key Laboratory of Parasite and Vector Biology, National Institute of Parasitic Diseases, Chinese Center for Disease Control and Prevention, Shanghai, China; ^6^ WHO Collaborating Centre for Tropical Diseases, National Center for International Research on Tropical Diseases, Shanghai, China; ^7^ Division of Endemic Disease Control and Prevention, Yingjiang County Center for Disease Control and Prevention, Yingjiang, China

**Keywords:** *Plasmodium vivax*, serological surveillance, post-elimination setting, anti-PvMSP-1 IgG, China

## Abstract

China was declared malaria free in June of 2021. In the post-elimination setting, vigilant surveillance is essential to sustain malaria free status. Serological surveillance has been recognized as an efficient tool for assessing the immunity levels and exposure risk in a population. In this study, a cross-sectional serological survey was conducted in Yingjiang County, China, in August–September, 2021. The study sites were villages along the borders with Myanmar, which have no local transmission since the last indigenous case registered in 2016. A total of 923 participants from six villages were enrolled. The majority was aged > 36 years (56.12%) and 12.46% (115/923) participants had experienced malaria infection at least once. A magnetic- bead-based assay was used to test antibodies against *Plasmodium vivax* antigen PvMSP-1_19_ to evaluate the prevalence of antibody positive subjects. A reversible catalytic model was used to assess the risk of exposure. The prevalence of anti-PvMSP-1_19_ IgG was 12.84% [95% confidence interval (CI): 9.22%–16.47%], 13.93% (95% CI: 10.11%–17.74%), and 3.57% (95% CI: 1.40%–5.75%) in three different line-of-defense areas, which differed significantly (*P* < 0.0001). The prevalence of anti-PvMSP-1_19_ IgG increased with age and no statistically significant difference was detected between the sexes. The reversible catalytic model indicated that the seropositive conversion rate and seronegative reversion rate were 0.0042, 0.0034, 0.0032 and 0.0024, 0.0004, 0.0065 in the first-, second-line-of-defense area and total areas, respectively, and the fitted value did not differ significantly from the observed value (*P* > 0.1). Although this study found the prevalence of antibody-positive subjects and the seroconversion rate in this post-elimination setting were lower than that in transmission setting, the population still had an exposure risk. Serological surveillance should be considered in post-elimination settings to provide valuable information with which to evaluate the risk of malaria re-establishment.

## 1 Introduction

Malaria remains a major public health problem. Despite the continued impact of COVID-19, the cases of malaria and death remained stable in 2021. According to the latest world malaria report, the global tally of malaria cases reached 247 million, with 619,000 deaths, in 2021 (WHO, 2022). The global malaria program has been very successful with 21 countries eliminating malaria since 2000 and 11 being certified malaria-free (Https://www.Who.Int/Activities/Treating-Malaria/Eliminating-Malaria, 2021). China launched the “National Action Plan for Malaria Elimination in China (2010–2020)” in 2010 (NHC, C 2010) and finally declared malaria free in June, 2021. Monitoring and surveillance are critical to the control and elimination of malaria, ensuring that progress and targets are accurately tracked, and thus supporting a reduction in the malaria burden, the elimination of the disease, and the prevention of its re-establishment (WHO, 2018; Hatherell et al., 2021). In a post-elimination setting, vigilant surveillance is vital for the prevention of the re-establishment of malarial transmission and the maintenance of a malaria-free status.

Measuring the prevalence of antimalarial antibody with serological markers is recognized as an efficient tool for assessing the immunity levels of a population and its exposure risk, which are good indicators of the intensity of malaria transmission in different malaria endemic settings (Stewart et al., 2009). Compared with traditional laboratory methods, serological indicators of malaria transmission intensity are highly sensitive and relatively robust to short term variations in transmission (Collins et al., 1968; Corran et al., 2007). Moreover, the persistence of antimalarial antibodies for long periods makes serological markers particularly suitable for surveillance in areas of low malaria transmission or no active transmission, or after its elimination (Drakeley et al., 2005; Corran et al., 2007; Dewasurendra et al., 2012).

Multiple serological assays have been developed and used to measure the transmission intensity of malaria. The immunofluorescence assay (IFA) was widely used in China during the 1980s–2000s (Tang et al., 1987; Sleigh et al., 1998) and the enzyme-linked immunosorbent assay (ELISA) has been the mainstay serological assay for a long time (Murungi et al., 2019). Recently, protein microarrays and magnetic-bead-based assays have been developed with characteristic high sensitivity, high specificity, and high throughput (Varela et al., 2018; Rogier et al., 2019).

The key areas in the national malaria elimination program, which were surveyed in the last step of the program, were the border areas between China and Myanmar, mainly in Yingjiang County. The last indigenous case of malaria in China was registered in this county in April 2016. In the last stage of the elimination program, a joint cross-border malaria prevention and control project termed “3+1 Strategy”, which was initiated for establishing a buffer zone of border malaria in these areas in 2017. Here, the “+ 1” was a catchment area in Myanmar side with a length of 20.5 km and a width of 2.5 km along the boundary, where the joint work and integrated measures were implemented, including funding and technical assistance, training on early case detection and management, focus response, vector control and surveillance, and health education, etc. The “3” referred to establishing three lines of defense in Yingjiang County where undertook national 1–3–7 approach to accelerate the process of malaria elimination (Lin et al., 2021). In this study, we conducted a cross-sectional study in Yingjiang County to estimate the prevalence of antimalarial antibodies and the exposure risk of the local population, to estimate the potential risk of the re-establishment of malaria in a post-elimination setting.

## 2 Materials and methods

### 2.1 Study sites

**Figure 1:**
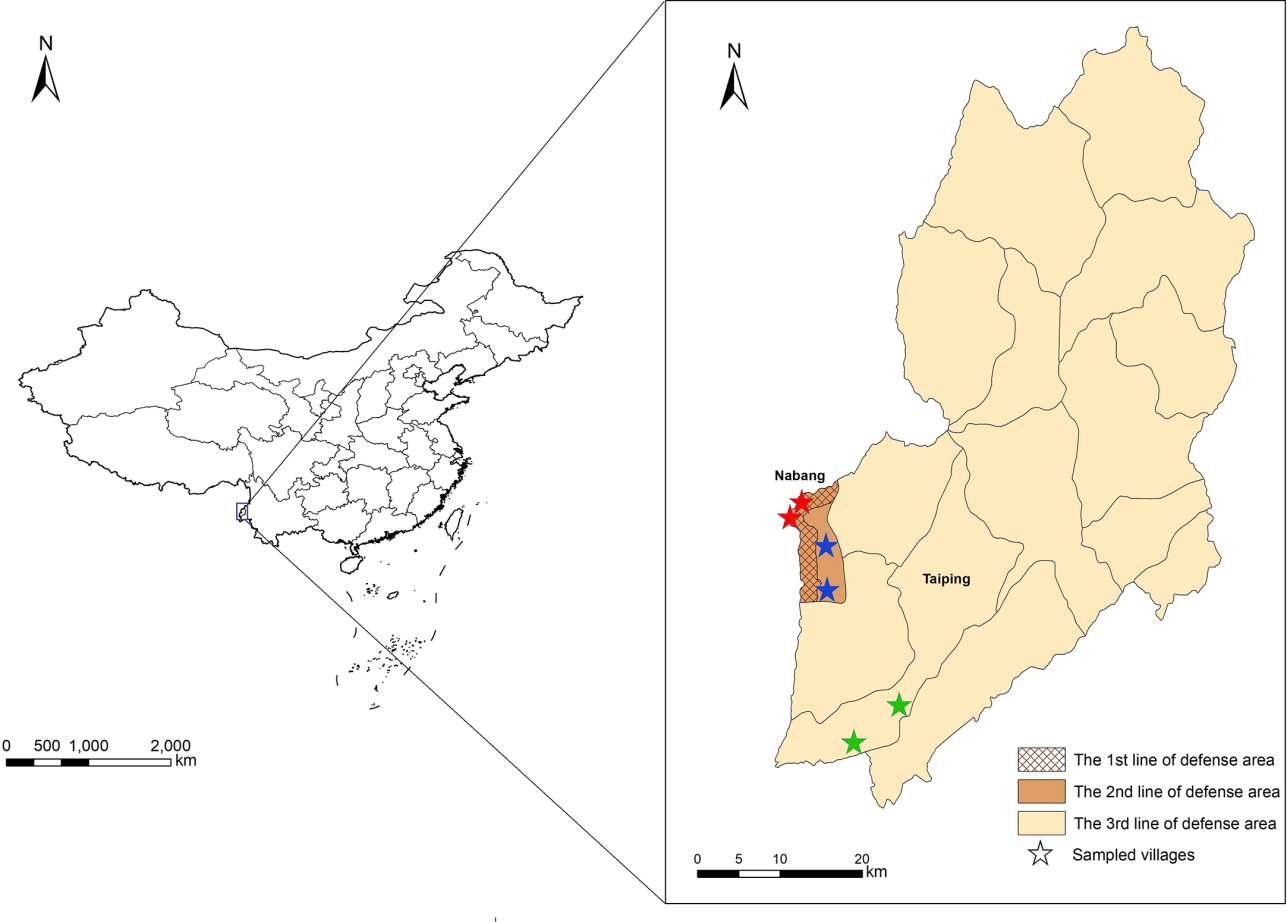
Locations of study sites and three different line-of-defense areas in Yingjiang County. Stars with red, blue, and green indicated villages sampled from the 1^st^, 2^nd^ and 3^rd^ line-of-defense areas.

This study was conducted in Yingjiang County, located in the west of Yunnan Province (**Figure 1**). Yingjiang is one of the 18 counties on the China-Myanmar border, in a subtropical monsoon climate zone, with an average annual temperature of 22.7°C and an annual rainfall of 2.65 m. Migration, plantation, and logging activities are frequent at the border (Zhang et al., 2016; Huang et al., 2021b). The vector *Anopheles minimus* is the dominant species of mosquito (Dong, 2000). Since the last indigenous case of malaria was reported in 2016, the majority of imported cases were *Plasmodium vivax* infections from Myanmar (Huang et al., 2021a).

In terms of the “3+1 Strategy”, three lines of defense were determined according to the potential risk of malaria re-establishment, and Yingjiang County was divided into three different areas. The first line-of-defense was defined as the border villages within 2 km of the boundaries in Nabang Township. The second line-of-defense was defined as non-border village 2 km apart in Nabang Township, and the third one was defined as other areas in the Yingjiang County (**Figure 1**). Villages were selected based on the principles of “3+1 Strategy” using stratified random sampling. Villages were first stratified into three categories based on the location in the first-, second-, and third-line-of-defense areas. Among each category or each line-of-defense area, two villages were sampled using simple random sampling method. In total, six villages were enrolled (**Figure 1**).

### 2.2 Sample size calculation

The seroconversion rate (SCR) was used as an indicator of the risk of malaria exposure in the population and sample sizes calculated for the different transmission settings were based on the value of SCR. In this study, as China was malaria free, the local parasite prevalence was defined as 0. Sample size was estimated based on previous study on size determination for estimating antibody seroconversion rate (Sepulveda and Drakeley, 2015), for a relative length of 1, which reported sample sizes varying from 67 (SCR=0.0324) to 248 (SCR=0.0036) in non-African settings given length of the 95% confidence interval for SCR. Here, we used the estimated SCR as 0.0036 with 95% accuracy, the minimum size of each line-of-defense area was 248 and total was 744.

### 2.3 Data and sample collection

After informed consent was obtained from all subjects, their demographic characteristics and behavioral and travel-related information were collected with a standardized questionnaire, using an E-data system. All data were uploaded and stored in a secure database at National Institute of Parasitic Diseases, China CDC.

Finger-prick blood was collected from all subjects for the diagnosis of malaria with a rapid diagnosis test (RDT) (Malaria HRP2/pLDH [P.f/Pan], Wondfo, Guangzhou, China). The finger-prick blood was also spotted onto filter papers (Whatman™ 903 and Whatman™ 3MM; GE Healthcare, Chicago, Illinois, USA), air-dried, and stored in individual sealed plastic bags with desiccant in a temperature-controlled facility until molecular and serological analysis.

### 2.4 Laboratory tests

#### 2.4.1 Parasite detection

*Plasmodium* DNA was extracted from the dried blood samples on 3MM filter paper with the QIAamp 96 DNA Blood Kit (Qiagen, Valencia, CA, USA), according to the manufacturer’s instructions. A real-time reverse transcription ultrasensitive polymerase chain reaction (PCR) analysis was performed following published methods, with an estimated limit of detection of 16 parasites/mL (Adams et al., 2015). The PCR results were interpreted based on the Ct values. If the results met the quality control conditions, samples with an S-shaped amplification curve and Ct values ≤ 35 were positive; samples with the Ct values > 38 or undetected were deemed to be negative; samples with values within the range 35 < Ct value ≤ 38 were retested. Only when a result was consistent, it was determined to be positive.

### 2.5 Serological tests

#### 2.5.1 Covalent coupling of antigen to beads

The recombinant blood-stage *P. vivax* malaria antigen PvMSP-1_19_ (R01601, Meridian Bioscience, Memphis, TN, USA), diluted in Phosphate Buffered Saline (PBS) to a concentration of 3.32 mg/mL, was used in the serological test. Carboxylated Luminex beads with different fluorescent moieties (Bio-Rad Inc., Hercules, CA, USA) were covalently coupled with bovine serum albumin (BSA) and the PvMSP-1_19_ antigen, using the modified protocol of Bio-Rad, as follows. The beads (5 × 10^6^ per assay) were resuspended in 80 μL of activation buffer (0.1 M NaH_2_PO_4_, pH 6.2) and activated with 10 μL of 1-ethyl-3-(3dimethylaminopropyl) carbodiimide hydrochloride (Pierce Biotechnology, Rockford, IL, USA) and N-hydroxysulfosuccinimide (50 mg/mL, Pierce Biotechnology). The activated beads were washed and resuspended in 100 μL of PBS (pH 7.4). The beads coupled beads with 8.0 μg of PvMSP-1_19_ antigen were resuspended in storage buffer and stored at 4°C in the dark.

### 2.6 Bead-based assay

The tested sera were isolated from blood spots eluted from 903 filter paper and diluted 1:4 before testing. Antigen-coated beads were thoroughly resuspended and diluted to a final concentration of 80 beads/μL. The secondary antibody (phycoerythrin-conjugated goat anti-human immunoglobulin G [IgG]; R&D Systems, Minneapolis, MN, USA), diluted 1:500 (1 μg/mL), was added and the beads were resuspended in 100 μL of PBS (pH 7.4) before analysis on Bio-Plex 200 system. The results were expressed as median fluorescence intensities (MFI). The cut-off value was defined as the MFI of the antibody in the negative control plus three standard deviations. Samples with an MFI value exceeding the cut-off value were determined as antibody positive.

### 2.7 Statistical analysis

All statistical analysis was performed with the statistical software SAS 9.4 (SAS Institute Inc, NC, USA). A χ^2^ test and Fisher’s exact test were used to evaluate the differences in seroprevalence among the different groups. A reversible catalytic model (Codeco, 2006) was used to assess the risk of exposure with the formula y = [*a*/(*a*+*b*)] × [1 − e^−(^ *^a^* ^+^ *^b^* ^)^ *^t^*], where *a* refers to the SCR from negative to positive; *b* refers to the SCR from positive to negative; and *t* is time. The seropositivity rates in the different defense areas were fitted according to subject age. The parameters of the seroconversion rate were estimated with Muench’s nomogram for the reversible catalytic model (Muench, 1959). The fitted antibody-positive rates of the different age groups in the villages were calculated. A correlation analysis was performed between the fitted antibody positive rate and the observed one. The goodness-of-fit was evaluated with a χ^2^ test (α =0.05) in GraphPad Prism 8.4.2 (GraphPad Software, LLC., San Diego, CA, USA). According to the parameters estimated with the best model, the curve-fitting equation for the seropositive rates in the three different line-of defense area was obtained, and the fitting curves for the different age groups and areas were plotted. Maps were created with ArcGIS 10.1 (Environmental Systems Research Institute, Inc., Redlands, CA, USA). A *P* value < 0.05 was deemed to indicate statistically significant differences.

## 3 Results

### 3.1 Demographic information

The field work was conducted in August–September, 2021. The total number of participants enrolled was 923, and the first-, second-, and third-line-of-defense areas contributed 327, 316, and 280 enrollees, respectively (**Table 1**). Six villages (two villages per line-of-defense area) were included and the sample sizes were 91–228 per village. All but one village (five villages) reported imported cases of malaria in 2019–2021 (**Supplementary File 1**).

**Table 1:** Demographic characteristics of participants from in three line-of-defense areas.

	The 1^st^ line-of-defense N (%)	The 2^nd^ line-of-defense N (%)	The 3^rd^ line-of-defense N (%)	Total N (%)
Sex				
Male	145 (44.34)	189 (59.81)	134 (47.86)	468 (50.70)
Female	182 (55.66)	127 (40.19)	146 (52.14)	455 (49.30)
Age (year)				
1–5	13 (3.98)	19 (6.01)	19 (6.79)	51 (5.53)
6–15	79 (24.16)	34 (10.76)	42 (15.00)	155 (16.79)
16–25	24 (7.34)	43 (13.61)	14 (5.00)	81 (8.78)
26–35	35 (10.70)	53 (16.77)	30 (10.71)	118 (12.78)
36–45	60 (18.34)	51 (16.14)	61 (21.79)	172 (18.63)
46–55	56 (17.13)	63 (19.94)	43 (15.36)	162 (17.55)
≥56	60 (18.35)	53 (16.77)	71 (25.36)	184 (19.93)
Past exposure to malaria				
Yes	51 (15.60)	37 (11.71)	27 (9.64)	115 (12.46)
No	266 (81.35)	279 (88.29)	250 (89.29)	795 (86.13)
No sure	10 (3.06)	0	3 (1.07)	13 (1.41)
Species of malaria infection				
*P. falciparum*	0	0	0	0
*P. vivax*	1^*^	0	0	1
Total	327	316	280	923

* One participant who had a history of travel to Myanmar was RDT-positive for P. vivax, and was deemed to be an imported case. In addition, this was also the only ultrasensitive PCR P. vivax positive one.

The ratio of females to males was 0.97:1. The majority of participants (56.12%, 518/923) were aged > 36 years. The age distribution differed slightly among the different areas, and the highest percentages were aged 6–15, 46–55, and 36–45 years in the first-, second-, and third-line-of-defense areas, respectively. Of the participants, 12.46% (115/923) had been exposed to malaria infection at least once in the past, and this value increased as subjects lived closer to the border. One participant who had a history of travel to Myanmar was RDT-positive for *P. vivax*, and was deemed to be an imported case. All the samples were tested with ultrasensitive PCR, and only one was *P. vivax* positive, the RDT-positive subject mentioned above.

### 3.2 Antibody prevalence

The numbers of participants showing *Plasmodium* antigen PvMSP-1_19_ IgG positive were 42, 44, and 10 in the first-, second-, and third-line-of-defense areas, respectively. The prevalence of anti-PvMSP-1_19_ IgG was 12.84% [95% confidence interval (CI): 9.22%–16.47%], 13.93% (95% CI: 10.11%–17.74%) and 3.57% (95% CI: 1.40%–5.75%), respectively. The prevalence of anti-PvMSP-1_19_ IgG differed significantly among three line-of-defense areas on a χ^2^ test and Fisher’s exact test (*P* < 0.0001). A paired analysis was used to determine the difference between each two pairs. There was no significant difference between the first- and second-line-of-defense areas (*P* > 0.05). When the first- and second-line-of-defense areas were combined and then compared with the third-line-of-defense area, the difference was significant (*P* < 0.0001).

### 3.3 Relationships between IgG-positive rate, sex, and age

**Figure 2:**
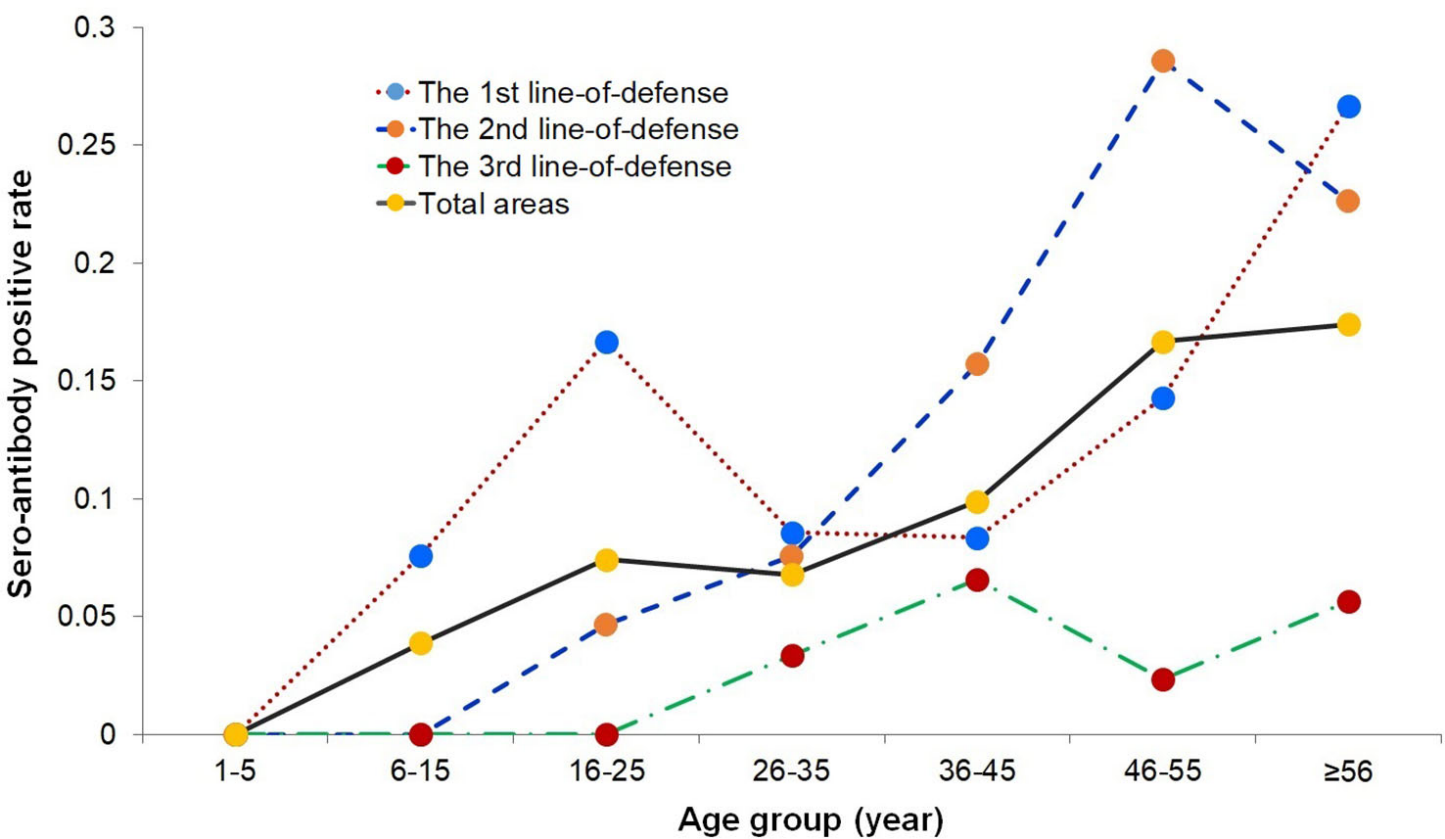
Seroprevalence rates in different age groups in the three line-of-defense area.

The prevalence of anti-PvMSP-1 19-IgG antibody-positive participants in male and female was 11.11% (52/468) and 9.67% (44/455), respectively, which did not differ significantly on a χ^2^ test (*P* > 0.05). When the differences between each line-of-defense area were analyzed separately, there was also no statistically significant difference (*P* > 0.05). The age-specific seropositive rates were calculated in the different line-of-defense areas (**Figure 2**). The seropositivity rates in the 1–5 year age group in the three line-of-defense areas were all 0. The prevalence of anti-PvMSP-1_19_ IgG positivity increased with age. The age group with the highest prevalence in the first- and second-line-of-defense areas and the total areas was > 45 years, whereas in the third-line-of-defense area, prevalence was highest in subjects aged 36–45 years and > 56 years.

### 3.4 Parameter estimation and goodness-of-fit test of reversible catalytic model

In terms of the conditions of the reversible catalytic model and the increasing trend in the age-specific seropositivity rates, the reversible catalytic model was fitted using the prevalence of IgG antibody positivity. The model indicated that the rate of conversion to seropositive were 0.0042, 0.0034, and 0.0032 in the first- and second-line-of-defense area and total areas, respectively, whereas the rate of reversion from seropositive to negative were 0.0024, 0.0004, and 0.0065, respectively (**Table 2**). The fitted antibody positivity rate for each age group in the different areas correlated with the observed antibody positive rate, and the correlation was best for the data from the whole area (R^2^ = 0.8582, *P* < 0.05). There was no significant difference between the fitted value using the reversible catalytic model and the observed value when the goodness-of-fit was evaluated with the χ^2^ test (*P* > 0.1).

**Table 2:** Estimated parameters and goodness-of-fit test using the reversible catalytic model.

Area	Rate of conversion to seropositive	Rate of reversion from seropositive to negative	R^2^	*P*	χ^2^	*P*
The 1^st^ line-of-defense	0.0042	0.0024	0.6176	0.0372	6.9804	>0.1
The 2^nd^ line-of-defense	0.0034	0.0004	0.8334	0.0041	8.8047	>0.1
Three line-of-defense areas (total areas)	0.0032	0.0065	0.8582	0.0027	8.7153	>0.1

**Figure 3:**
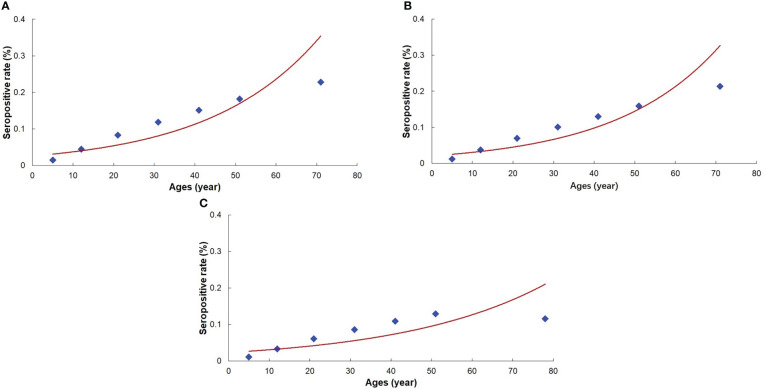
Fitting curve for the prevalence of *Plasmodium vivax* antibody. **(A)**: The 1^st^ line-of-defense area. **(B)**: The 2^nd^ line-of-defense area. **(C)**: Three line-of-defense areas (Total areas).

The curve fitting equation for the antibody positive rate was obtained using the parameters estimated with the reversible catalytic model (**Supplementary File 2**), and the fitting curve for the antibody positive rate indicated that the reversible catalytic model fitted well (**Figure 3**).

## 4 Discussion

Serological surveillance of malaria to monitor exposure, transmission, and immunity involves the use of *Plasmodium*-species-specific antibodies as biomarkers, and has significant potential utility in enhancing the effectiveness of malaria control and elimination programs (Elliott et al., 2014). Seroprevalence reflects the cumulative exposure of a population to malaria and is therefore believed to be less affected by seasonality and/or unstable transmission than other parameters, making it more suitable for use in predicting variations in transmission (Drakeley et al., 2005; Bousema et al., 2010). As one serological surveillance tool, IFA had been used historically as part of malaria control programs in China for a long time (Tang et al., 1987; Duo-Quan et al., 2009). However, serological surveillance has not been used in the elimination and post-elimination phases since the last indigenous cases of malaria were recorded in 2016. In the present study, a cross-sectional serological survey was conducted to estimate the prevalence of *P. vivax* antibodies and the exposure risk in Yingjiang County, which poses the highest risk of malaria re-establishment in China. This study was the first serological evaluation in malarial post-elimination phase in China, and provided valuable information on the potential re-establishment risk in a post-elimination setting.

Antigen-specific antibodies can reflect both past and present exposure to *Plasmodium* parasites, rather than presenting just a single snapshot in time. Antibodies are generated during infection and certain antibodies can be detected even after the infection has been cleared or the transmission season is over. The candidate antigens of *P. vivax* used for serological surveillance mainly include the *P. vivax* reticulocyte binding proteins (RBPs), the erythrocyte binding protein (EBP), the merozoite surface proteins (MSPs), apical membrane antigen 1 (AMA1), glutamate-rich protein-R0 (GLURP-R0), and liver-stage antigen 1 (LSA-1). PvMSP-1_19_ is the most immunogenic antigen of *P. vivax* evaluate the cumulative exposure risk in an investigated population (Del Portillo et al., 1991; Fernandez-Becerra et al., 2010; Helb et al., 2015), which is a suitable marker for the serological surveillance of malaria in low- or no-malarial-transmission settings.

Our findings indicated that the prevalence of PvMSP-1_19_ antibody positivity did not differ significantly between the first- and second-line-of-defense areas, whereas it did differ significantly (*P* < 0.0001) among three line-of-defense areas. These results may be attributable to differences in the exposure risk and geographic locations of these areas, because the villages from the first- and second-line-of-defense areas were in Nabang Township, adjacent to Laiza City, Kachin State, Myanmar, which reported thousands of cases of malaria annually (Zhang et al., 2016; Huang et al., 2021a). There is one provincial port and several boundary passages in this township and hundreds of migrants cross the border every day. Some sections of the border comprise a stream that is only a few meters wide, and mosquitoes carrying parasites can cross the border easily. These factors caused the higher risk of exposure to malaria in the populations in the first- and second-line-of-defense areas than in the other township in the third-line-of-defense area, which does not border Myanmar.

In this study, the prevalence of PvMSP-1_19_ antibody positivity was not associated with sex. However, its distribution across age groups differed and the majority of the antibody-positive population was in the age group > 36 years (36–45, 46–55, and > 56 years), whereas no children aged 1–5 years were antibody positive. The seroprevalence in adults reflected the exposure to infection over a longer time period (Ashton et al., 2011; Yukich et al., 2013; Alemu et al., 2014) and several factors could contribute to this outcome as well. First, population movement is one challenge for malaria control and elimination, especially in border areas (Martens and Hall, 2000). Population movement from transmission area to non-malaria-endemic areas can result in imported infections or corresponding increasing seroprevalence in these migrant population. Secondly, although ultrasensitive detection methods could detect subclinical malaria infections with low parasitemia, hidden indigenous transmission and asymptomatic infections might be existing (Egger et al., 2022), which would be identified as seropositive using serological test. Third, as the unique biological characteristics of *P. vivax* with residual dormant stages in the liver and periodic relapses (Adams and Mueller, 2017), population with historical *P. vivax* infection might lead to relapse and seropositivity over a prolonged period. While, the seropositivity in younger children could reflect the recent malaria exposure rates and transmission intensities. The prevalence of antibodies in children aged 1–5 years in three line-of-defense areas were all zero, which strongly supported the indigenous malaria transmission has been suppressed in recent years in the study areas. Moreover, the fitting curve based on the age-specific antibody positivity rates also predicted that the exposure risk of malaria in the 1–5 year age group was 0, which proved the absence of local transmission in the malaria elimination phase, and provided a serological basis for the local confirmation and consolidation of malaria elimination. A similar study in Sri Lanka based on a serological survey after the declaration of malaria elimination also showed that seropositivity in the child group was 0 (Dewasurendra et al., 2017).

In this study, the results showed that the prevalence of PvMSP-1_19_ antibody positivity increased with age in all the study areas. The risk of malarial exposure predicted with the reversible catalytic model showed that the seroconversion rates in the first- and second-line-of-defense areas were 0.0042 and 0.0034, respectively. The predicted rate was much lower than in other studies that used the same serological marker, PvMSP-1_19_. Two studies undertaken in Cambodia in 2005 and 2011 found seroconversion rates of 0.184 in 2005 and 0.011 in 2012 (Cook et al., 2012; Kerkhof et al., 2016). Another serological study conducted in Yingjiang County in 2014 showed a seroconversion rate of 0.0087 (Yao et al., 2022), which was also higher than our findings. This reduced seroconversion rates also reflected the outcome and effectiveness of the malaria elimination measures in this area.

Although this serological survey showed that seroconversion rates were lower in a post-elimination setting, there was still a risk of exposure to malaria among the populations in the first- and second-line-of-defense areas. When moving into the elimination or post-elimination phase, local transmission is zero, but the potential risk of malaria re-establishment does not disappear, especially in border areas where malaria is still endemic on the other side of the border. Migrant population crossing the border and the lack of barriers to malarial vectors are also challenges to the maintenance of a malaria-free status. Although great achievements have been made in eliminating indigenous cases of malaria in China, the elimination campaign still faces challenges in regions close to international borders (Huang et al., 2021b). It is difficult to maintain a malaria-free status in the post-elimination setting, and the risk of its re-establishment is plausible if the surveillance systems, malaria vigilance, and personnel for the diagnosis and treatment of malaria are inadequate (Schapira and Kondrashin, 2021). Therefore, compared with traditional serological methods, this high-throughput and sensitive serological surveillance technique should have significant utility in evaluating the risk of malaria re-establishment in post-elimination settings.

## 5 Limitations

This study had several limitations. First, although multiple serological assays have been used to measure the transmission of *P. vivax*, no standardized serological marker is currently available. In this study, only one antigen, *P. vivax* antigen PvMSP-1_19_, was used in the serological test. Second, the catalytic model was used at the population level, so individual information and other related factors were ignored. Third, the study sites were only selected in border areas with a potentially high risk of re-establishment.

## 6 Conclusion

This is the first serological report to estimate the exposure risk of a population in the post-elimination phase of malaria in China. It indicated that the prevalence of anti-malaria antibody positivity was relatively low in the post-elimination setting, although an exposure risk persisted. Serological surveillance should be considered in the post-elimination setting to provide valuable information about the potential re-transmission risk in post-elimination settings.

## Data availability statement

The original contributions presented in the study are included in the article/**Supplementary Material**. Further inquiries can be directed to the corresponding authors.

## Ethics statement

The studies involving human participants were reviewed and approved by National Institute of Parasitic Diseases, Chinese Center for Disease Control and Prevention. Written informed consent to participate in this study was provided by the participants’ legal guardian/next of kin.

## Author contributions

FH and Z-GX conceived and designed the study; FH and YC carried out the data analysis. YC and SW conducted the laboratory work. SL and XRG collected the field samples. FH drafted the initial manuscript. ZH and XG contributed to data review and manuscript revision. All authors contributed to the article and approved the submitted version. 
