# Supplementary material for: Serological surveillance on potential Plasmodium vivax exposure risk in a post-elimination setting
Source: Front Cell Infect Microbiol. 2023 Mar 9;13:1132917. doi: 10.3389/fcimb.2023.1132917 (PMC10034364; doi:10.3389/fcimb.2023.1132917)
Supplement: Supplementary file 1 [file Table_1.docx]

**Table S1 Numbers and species of imported cases of malaria in sampled villages in 2019–2021**

| **Villages** | **2019** | | | **2020** | | | **2021** | | | **Total** |
| --- | --- | --- | --- | --- | --- | --- | --- | --- | --- | --- |
|  | *P. vivax* | *P. falciparum* | Mix infection | *P. vivax* | *P. falciparum* | Mix infection | *P. vivax* | *P. falciparum* | Mix infection |  |
| Shangtianba | 0 | 0 | 0 | 2 | 0 | 0 | 2 | 0 | 0 | 4 |
| Jiedao | 2 | 0 | 0 | 4 | 0 | 0 | 9 | 0 | 0 | 15 |
| Daonong | 0 | 0 | 0 | 0 | 0 | 0 | 1 | 0 | 0 | 1 |
| Qiaotou | 7 | 0 | 0 | 4 | 0 | 0 | 5 | 0 | 0 | 16 |
| Xueli | 10 | 0 | 0 | 6 | 0 | 0 | 3 | 0 | 0 | 19 |
| Gecun | 0 | 0 | 0 | 0 | 0 | 0 | 0 | 0 | 0 | 0 |
| Total | 19 | 0 | 0 | 16 | 0 | 0 | 20 | 0 | 0 | 55 |
