# Supplementary material for: Serological surveillance on potential Plasmodium vivax exposure risk in a post-elimination setting
Source: Front Cell Infect Microbiol. 2023 Mar 9;13:1132917. doi: 10.3389/fcimb.2023.1132917 (PMC10034364; doi:10.3389/fcimb.2023.1132917)
Supplement: Supplementary file 2 [file Table_2.docx]

**Table S2 The curve fitting equation for the antibody positive rate in the three line-of-defense area using the reversible catalytic model**

| **Area** | **Rate of reversion to seropositive** | **The curve fitting equation** |
| --- | --- | --- |
| The first-line-of-defense | 0.0042 | y=0.6283*(1-e^-0.0067*t^) |
| The second-line-of-defense | 0.0034 | y=0.8879*(1-e^-0.0038*t^) |
| Three line-of-defense areas (total areas) | 0.0032 | y=0.3322*(1-e^-0.0096*t^) |
